# Real-time analysis of the diphtheria outbreak in forcibly displaced Myanmar nationals in Bangladesh

**DOI:** 10.1186/s12916-019-1288-7

**Published:** 2019-03-12

**Authors:** Flavio Finger, Sebastian Funk, Kate White, M. Ruby Siddiqui, W. John Edmunds, Adam J. Kucharski

**Affiliations:** 1 Centre for the Mathematical Modelling of Infectious Diseases, London School of Hygiene and Tropical Medicine, London, UK; 2 Médecins Sans Frontières, Amsterdam, Netherlands; 3 Médecins Sans Frontières, London, UK

**Keywords:** Diphtheria, Real-time modelling, Bangladesh, Refugees, Infectious disease, Epidemiological modelling, Mathematical modelling, Health in humanitarian crises

## Abstract

**Background:**

Between August and December 2017, more than 625,000 Rohingya from Myanmar fled into Bangladesh, settling in informal makeshift camps in Cox’s Bazar district and joining 212,000 Rohingya already present. In early November, a diphtheria outbreak hit the camps, with 440 reported cases during the first month. A rise in cases during early December led to a collaboration between teams from Médecins sans Frontières—who were running a provisional diphtheria treatment centre—and the London School of Hygiene and Tropical Medicine with the goal to use transmission dynamic models to forecast the potential scale of the outbreak and the resulting resource needs.

**Methods:**

We first adjusted for delays between symptom onset and case presentation using the observed distribution of reporting delays from previously reported cases. We then fit a compartmental transmission model to the adjusted incidence stratified by age group and location. Model forecasts with a lead time of 2 weeks were issued on 12, 20, 26 and 30 December and communicated to decision-makers.

**Results:**

The first forecast estimated that the outbreak would peak on 19 December in Balukhali camp with 303 (95% posterior predictive interval 122–599) cases and would continue to grow in Kutupalong camp, requiring a bed capacity of 316 (95% posterior predictive interval (PPI) 197–499). On 19 December, a total of 54 cases were reported, lower than forecasted. Subsequent forecasts were more accurate: on 20 December, we predicted a total of 912 cases (95% PPI 367–2183) and 136 (95% PPI 55–327) hospitalizations until the end of the year, with 616 cases actually reported during this period.

**Conclusions:**

Real-time modelling enabled feedback of key information about the potential scale of the epidemic, resource needs and mechanisms of transmission to decision-makers at a time when this information was largely unknown. By 20 December, the model generated reliable forecasts and helped support decision-making on operational aspects of the outbreak response, such as hospital bed and staff needs, and with advocacy for control measures. Although modelling is only one component of the evidence base for decision-making in outbreak situations, suitable analysis and forecasting techniques can be used to gain insights into an ongoing outbreak.

**Electronic supplementary material:**

The online version of this article (10.1186/s12916-019-1288-7) contains supplementary material, which is available to authorized users.

## Background

Between August and December 2017, more than 625,000 Rohingya fled into Bangladesh as a result of large-scale operations conducted by the Myanmar military in Rakhine state [1]. This resulted in one of the largest refugee crises in recent history. The new refugees joined more than 212,000 Rohingya already present from past exoduses and settled in mostly informal makeshift camps and amongst the host community [2]. The poor living conditions typically seen in refugee settings—such as reduced access to health care; low standards of water, sanitation and hygiene; malnutrition and high population density—are often associated with infectious disease outbreaks. Such settings can enable transmission of infections associated with poor water and sanitation, such as cholera and hepatitis E [3, 4], as well as infections that in other settings are prevented through routine childhood vaccination, such as measles and diphtheria [5].

On 10 November, a case of diphtheria was reported to a health care facility in Balukhali run by Médecins sans Frontières (MSF). Diphtheria is caused by the diphtheria toxin-producing bacterium *Corynebacterium diphtheriae*, which is transmitted through droplets and close physical contact*,* typically resulting in disease of the upper respiratory tract. Symptoms can include the formation of a pseudo-membrane obstructing airways or markedly enlarged lymph nodes. Common complications include difficulty breathing and swallowing and myocarditis. The incubation period is typically between 2 and 5 days (range 1–10) [6], with an estimated basic reproduction number of 4–5 [7]. Due to its high transmissibility and reported case fatality rates of over 10% [6], diphtheria was a worldwide major public health concern with one million cases and 50,000 to 60,000 deaths per year in the 1970s, leading to the inclusion of diphtheria toxoid-containing vaccines in the Expanded Programme on Immunization by the World Health Organization (WHO). As a result, the global diphtheria incidence has decreased drastically in the second half of the last century (by over 90% between 1980 and 2000), but remains of significant concern in areas with low vaccination coverage [6]. Recently, outbreaks have occurred in Yemen, Venezuela, Indonesia and Haiti [8, 9].

**Fig. 1:**
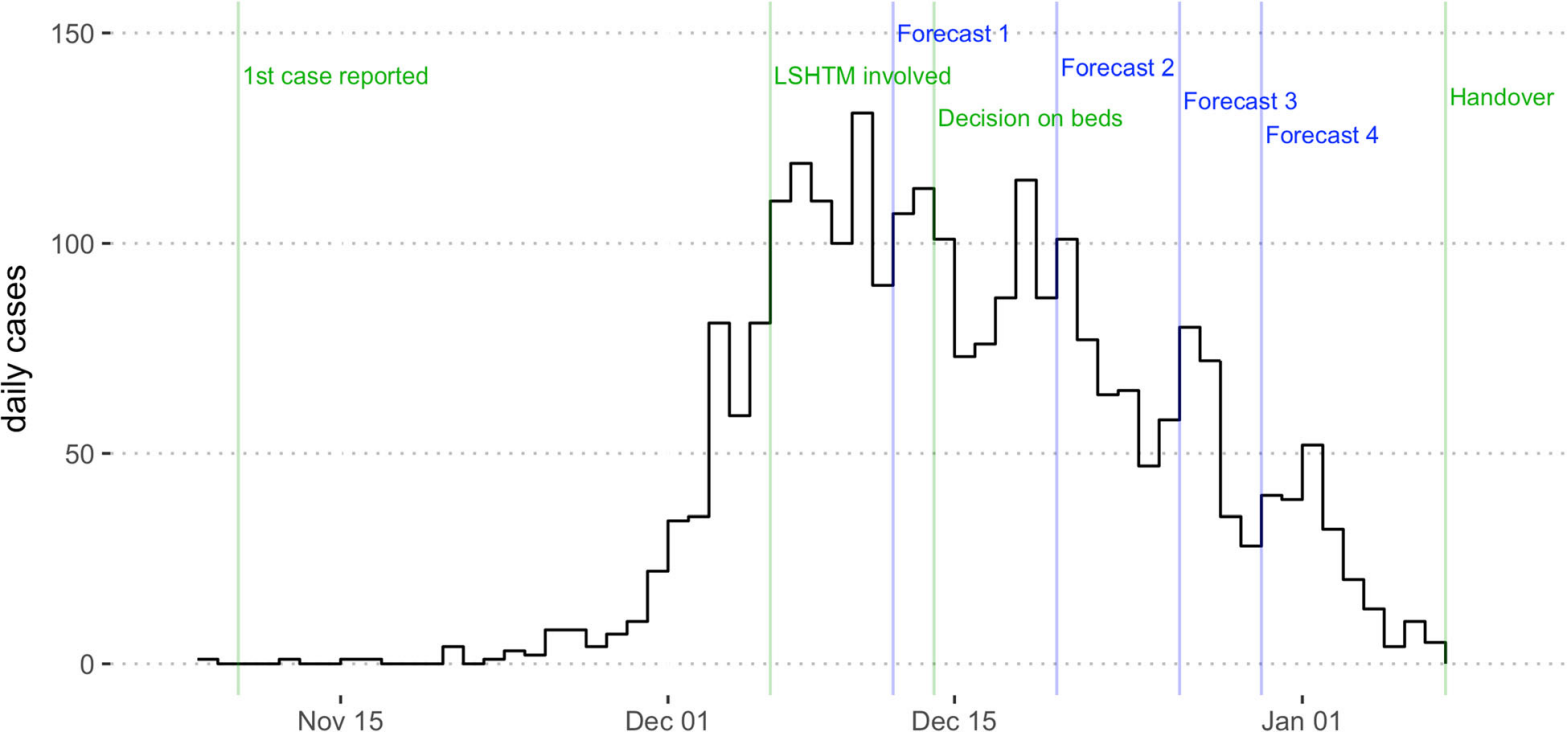
Outbreak analysis timeline with respect to the epidemic curve (black line). Green lines show the timing of events relevant to analysis: reporting of the first case, involvement of modellers at LSHTM, MSF decision on bed numbers required and MSF handover of the treatment centre. Blue lines show the date on which each of the four LSHTM forecasts was communicated to MSF

In the month after the first case was reported in Balukhali, there were 440 additional suspected cases reported in nearby refugee settlements, 168 of which were reported on 9 Dec 2017 alone. An initial temporary Diphtheria Treatment Centre (DTC) in Balukhali run by MSF opened in the week starting on 17 December (epidemiological week 51). In the early stages of an infectious disease outbreak, it is crucial to understand the epidemiology of the infection. By quantifying transmission dynamics, it is possible to produce forecasts of future incidence [10, 11] and evaluate the potential impact of control measures [12, 13]. The significant rise in the number of diphtheria cases in early December (Fig. 1) led to the establishment of a collaboration between teams from MSF and the London School of Hygiene and Tropical Medicine (LSHTM) with the goal to forecast the potential scale of the outbreak and the resulting resource needs using transmission dynamic models. The first forecast was issued on 12 December, with three more subsequently issued, before the DTC in Balukhali closed on 8 January 2018 (handing over diphtheria activities to several newly opened DTCs run by different organizations). Such analysis can face multiple challenges in real time, including delays and variability in available data streams, limited pre-existing epidemiology studies and knowledge gaps about risk factors and immunity in the host population. Besides describing the modelling methodology and forecasts, we report on the practical implications of the analysis, examining the role real-time modelling of infectious disease dynamics can play in operations and decision-making in a complex humanitarian crisis.

## Methods

### Data

Between 8 November and 31 December 2017, a total of 2624 cases (495 from Kutupalong (attack rate 0.12%), 1868 from Balukhali (attack rate 0.97%) and 261 from other or unknown nearby locations) presented at the Diphtheria Treatment Centre in Balukhali run by MSF. The total refugee population had been estimated at around 608,000 in early December (Additional file 1). From 9 December 2017 to 12 January 2018, we received daily anonymized line lists of suspected cases seen at this centre (Additional file 2). Fields included the patient identification number, sex, age, approximate address of the patient, date of onset of symptoms, date of reporting to the DTC, signs and symptoms, treatment and clinical outcome. First, we checked the line list for objectively erroneous values—such as dates that were in the future or dates of reporting prior to the date of onset of symptoms—and corrected these where possible. We then computed the daily crude incidence within three age groups (0–4, 5–14 and ≥ 15 years) and two geographical locations (Balukhali and Kutupalong, with other/unknown locations omitted from the analysis).

### Adjustment for delayed reporting

To adjust for delays between symptom onset and case presentation and estimate the actual incidence at a given point in time, we computed the cumulative distribution of reporting delays of reported cases (defined as the number of days between symptom onset and case presentation). We then divided the crude daily incidence values by the corresponding values in this distribution (e.g. delay 0 for the current day, delay 1 for the previous day etc.) to obtain the adjusted incidence. Initially, we used all previously reported cases to compute the delay distribution, but changed the analysis window as more data became available. From 18 December onwards, we used cases with symptom onset between 10 December and 16 December to compute the delay distribution; from 24 December, we used cases with symptom onset between 17 December and 23 December, and starting on 30 December, we used all cases with symptom onset since 10 December.

### Mathematical model and forecasting

We modelled diphtheria transmission dynamics using an age- and location-stratified deterministic compartmental model, which followed a susceptible-exposed-infective-recovered (SEIR) structure. Upon exposure to infection, initially susceptible hosts (*S*) transitioned to a latent compartment (*E*), then an infectious compartment (*I*) and finally a recovered and immune compartment (*R*). We included three age groups in the model (denoted by subscript *a*): aged under 5 (denote by subscript 1), aged 5 to 14 (subscript 2) and aged over 14 (subscript 3). We also modelled two locations (denoted by subscript *L*): Balukhali and Kutupalong. We assumed that proportion *r*_*L*_ of cases were reported and a proportion *h* of reported cases would require inpatient treatment. We further assumed the time taken to seek treatment, *T*_1_, as well as the average duration of hospital stay, *T*_2_. We did not include births and deaths in the model as the average human lifespan was much longer than the duration of the outbreak.

$$ {\displaystyle \begin{array}{lll}\frac{dS_{a,L}}{dt}& =& -\Lambda {S}_{a,L}\\ {}\frac{dE_{a,L}}{dt}& =& {\Lambda}_L{S}_{a,L}-{vE}_{a,L}\\ {}\frac{dI_{a,L}}{dt}& =& {vE}_{a,L}-\gamma {I}_{a,L}\\ {}\frac{dR_{a,L}}{dt}& =& \gamma {I}_{a,L}\\ {}\frac{d{\Lambda}_L}{dt}& =& \sum \limits_a{I}_{a,L}\\ {}\frac{dC_{a,L}}{dt}& =& \gamma {I}_{a,L}\\ {}\frac{dH_L}{dt}& =& {\tau}_1h\kern0ex {r}_L\sum \limits_a{I}_{a,L}-{\tau}_2{H}_L\end{array}} $$

Here, Λ_*L*_ denotes the force of infection for camp *L* and *C*_*a*,*L*_ the cumulative number of cases. We fixed the mean latent period, 1/*v*, and infectious period, 1/*γ*, and estimated the camp-specific transmission rate, *β*_*L*_. We also estimated the initial number of infective individuals, ${I}_{a,L}^0$, for each age group and location. We assumed the initial proportion susceptible in the 5–14 age group, ${S}_{2,L}^0$, with susceptibility in the other two age groups relatively lower, i.e. ${S}_{1,L}^0={\alpha}_{1,L}\kern0ex {S}_{2,L}^0$, and where ${S}_{3,L}^0={\alpha}_{3,L}\kern0ex {S}_{2,L}^0$ were parameters to be fitted.

The gaps in time between the onset dates of early reported cases suggested the generation time of infection may have been around 4–5 days (Additional file 3: Figure S1). We therefore assumed an incubation period of 3 days and an infectious period of 3 days. Averaging over hosts who have completed their infectious period, this implied an expected generation time of 4.5 days. However, the generation time across everyone currently infected early in an epidemic may be shorter, as most hosts will have been infected by people early in their infectious period, shrinking the generation time. Vaccination coverage for DPT3 in Myanmar was reported to be 85% in 2012 [14], but coverage was likely to be lower in the Rohingya population [15]. In November 2017, a health survey performed by MSF estimated that measles vaccination coverage in Rohingya children aged between 6 and 59 months was 20–25%, following a vaccination campaign in children between 9 months and 14 years old [16]. Vaccination data for diphtheria were not available, but we assumed that 20% of the 5–14 age group were initially immune to diphtheria.

Besides the fixed parameters, the model had seven parameters that were estimated independently for each location: the proportion of cases reported, the transmission rate, the relative susceptibility in under 5 and over 14 age groups and the initial proportion of infectious in each age group. When an outbreak is growing exponentially and with the limited data available initially, it is often not possible to estimate the initial number of infectious cases and the proportion of cases reported separately with non-informative priors. Generally, these two parameters are inversely correlated; the same issue occurs for the initial level of susceptibility and the transmission rate. We therefore assumed that the initial proportion of susceptibles in the middle age group was 80% (equivalent to a Dirac delta prior). To obtain a prior distribution for the proportion of reported cases, we performed a rough calculation using data from the pre-vaccination era. Prior to widespread DTP3 vaccination coverage in the UK, there were around 55,000 cases of diphtheria per year [17] and 750,000 live births each year [18]. Given that diphtheria has a relatively high basic reproduction number, *R*_0_, of 4–5, almost all initially susceptible individuals would be expected to eventually become infected in the absence of vaccination [7]. Hence, almost all newborns would become infected at some point. Based on the data from the pre-vaccination era, this would suggest that at least 7% (55,000/750,000) of all diphtheria infections appear as cases. We therefore imposed a strong gamma prior distribution on the proportion of cases reported, which had a mean of 10% and a standard deviation of 2.2%. We assumed uniform positive priors on all other parameters, with range (0, 1) for the relative susceptibility of the under 5 and over 14 age groups and the proportion of cases reported.

**Fig. 2:**
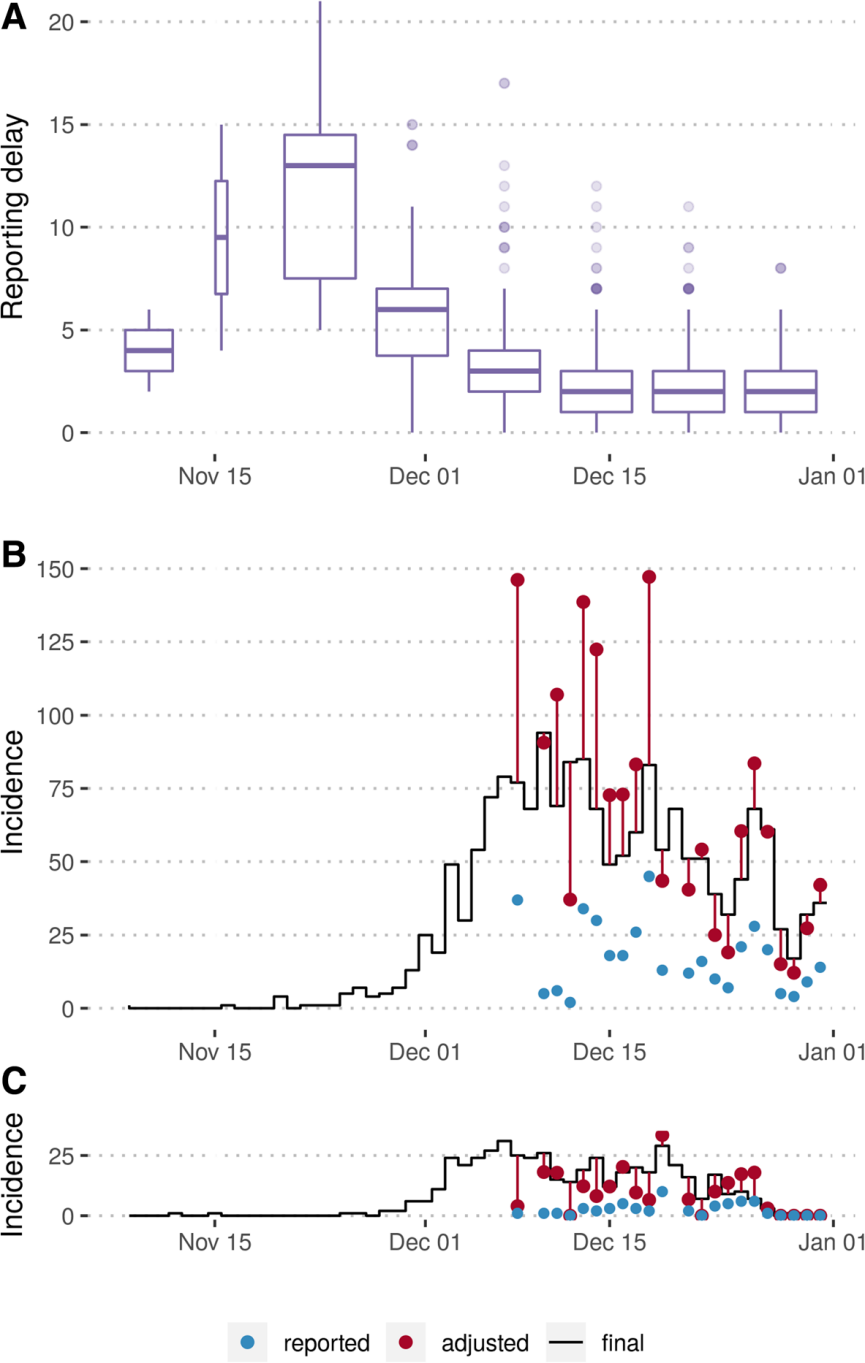
Adjustment for the delay between symptom onset and case presentation (reporting delay). Evolution of the reporting delay (vertical axis) by week (horizontal axis) (**a**). Daily incidence of diphtheria cases in Balukhali (**b**) and Kutupalong (**c**) as reported within the first day after symptom onset (blue dots), adjusted for reporting delays (red dots) and as seen retrospectively (black line, data from 12 January 2018)

The 14 free parameters in the model (seven for each camp) were calibrated to the adjusted incidence at each location using a Markov Chain Monte Carlo (MCMC) model fitting procedure via a Metropolis-Hastings algorithm. Incidence was adjusted for reporting delays (see previous section), and the most recent date of reported cases in each dataset was removed when fitting, as these data were most likely to be biased by delays (Fig. 2b, c). The case count in age group *α* in camp *L* for week *t* was defined as *c*_*t*, *a*, *L*_ = *C*_*t*, *a*, *L*_ − *C*_*t* − 1, *a*, *L*_. We assumed that reported weekly cases followed a negative binomial distribution with mean *r*_*L*_*C*_*t*, *a*, *L*_ and a camp-specific dispersion parameter *ϕ*_*L*_ to account for the potential for temporal variability in reporting between weeks [19]. The statistical and mathematical models were implemented in R version 3.4.3 [20] using the deSolve package [21].

As the forecasting analysis was developed from scratch in real time, the modelling framework was continuously refined to improve efficiency of mixing and integration with data cleaning and processing pipelines. In real time, we ran MCMC chains for between 20,000 and 100,000 iterations, with lengths chosen to ensure convergence of model forecasts. In the analysis presented here, we show results based on outputs from three chains of 100,000 iterations using the final version of the forecasting model, with a burn-in period of 20,000 iterations. The marginal distributions of posterior estimates are shown in Additional file 3: Figures S2–S5, with MCMC trajectories in Additional file 3: Figures S6–S9. Key parameters of interest (*R*_0_, proportion reported, relative susceptibility) all had an effective sample size of at least 100. A summary of all parameters and their prior distributions is given in Table 1.

**Table 1:** Parameters used in the model. Parameters that are camp-specific take independent values for Balukhali and Kutupalong, and the initial proportion of infectious people is specific to each age group in each camp

Name	Description	Age or camp specific?	Value	Prior distribution
1/*v*	Incubation period	No	3 days	
1/γ	Infectious period	No	3 days	
*β*_*L*_	Transmission rate	Camp	Fitted	Uniform(0,*∞*)
*r*_*L*_	Proportion of cases reported	Camp	Fitted	Gamma(20,0.005)
*ϕ*_*L*_	Dispersion parameter for reporting	Camp	Fitted	Uniform(0,*∞*)
α_1, *L*_	Relative susceptibility in under 5 age group	Camp	Fitted	Uniform(0,1)
α_3, *L*_	Relative susceptibility in over 15 age group	Camp	Fitted	Uniform(0,1)
${S}_{2,L}^0$	Initial proportion susceptible in the age group 5–14 years	No	0.80	
${I}_{a,L}^0$	Initial proportion of infectious	Camp and age	Fitted	Uniform(0,*∞*)

The model was used to generate forecasts of future incidence on 12 December, 20 December, 26 December and 30 December. To produce a forecast, the model was calibrated to the past adjusted incidence in each age group and location and 1000 epidemics simulated up to 2 weeks into the future. Median and uncertainty (95% posterior predictive interval (PPI)) were communicated to partners. When forecasting bed requirements, we assumed that 15% of the reported cases would require treatment as inpatients, and an average hospital stay would be 5 days. These estimates were informed by early patient data in the line list.

## Results

### Adjustment for delayed reporting

The delay between symptom onset and case presentation was 2 and 6 days respectively for the first two reported cases. This subsequently increased to 13 (range 5 to 21) days before stabilizing around a median value of 2 (range 0 to 12) days from early December onwards (Fig. 2a). Comparing estimated incidence adjusted for reporting delays with the final incidence subsequently reported, we found that our method reduced the bias introduced by delayed reporting, although results showed high variability (Fig. 2b, c).

Between 8 November and 31 December, medians of 31% (95% PPI 4–48%) of cases were reported within 1 day of symptom onset (i.e. until the end of the first day after onset), 61% (95% PPI 22–82%) within 2 days and 84% (95% PPI 45–94%) within 3 days.

Adjusting for delayed reporting, we overestimated the actual daily incidence by a median of 7% (95% PPI − 61 to 46%) from the unadjusted incidence known within 1 day after symptom onset, by 5% (95% PPI − 27 to 52%) from the unadjusted incidence known 2 days after onset and by 0% (95% PPI − 17 to 40%) from the unadjusted incidence known 3 days after onset. Note that negative percentages indicate an underestimation of the actual incidence here.

### Forecasts

We generated four sequential forecasts of future incidence, each stratified by age group and location. The initial forecast was produced and communicated to MSF field staff on 12 December. Subsequent forecasts were issued on 20, 26 and 30 December. Because model fitting and data processing were continuously being refined in real time, with a focus on outbreak response rather than detailed evaluation, here, we present results from the final version of the model to evaluate how well such forecasting methods can work.

**Fig. 3:**
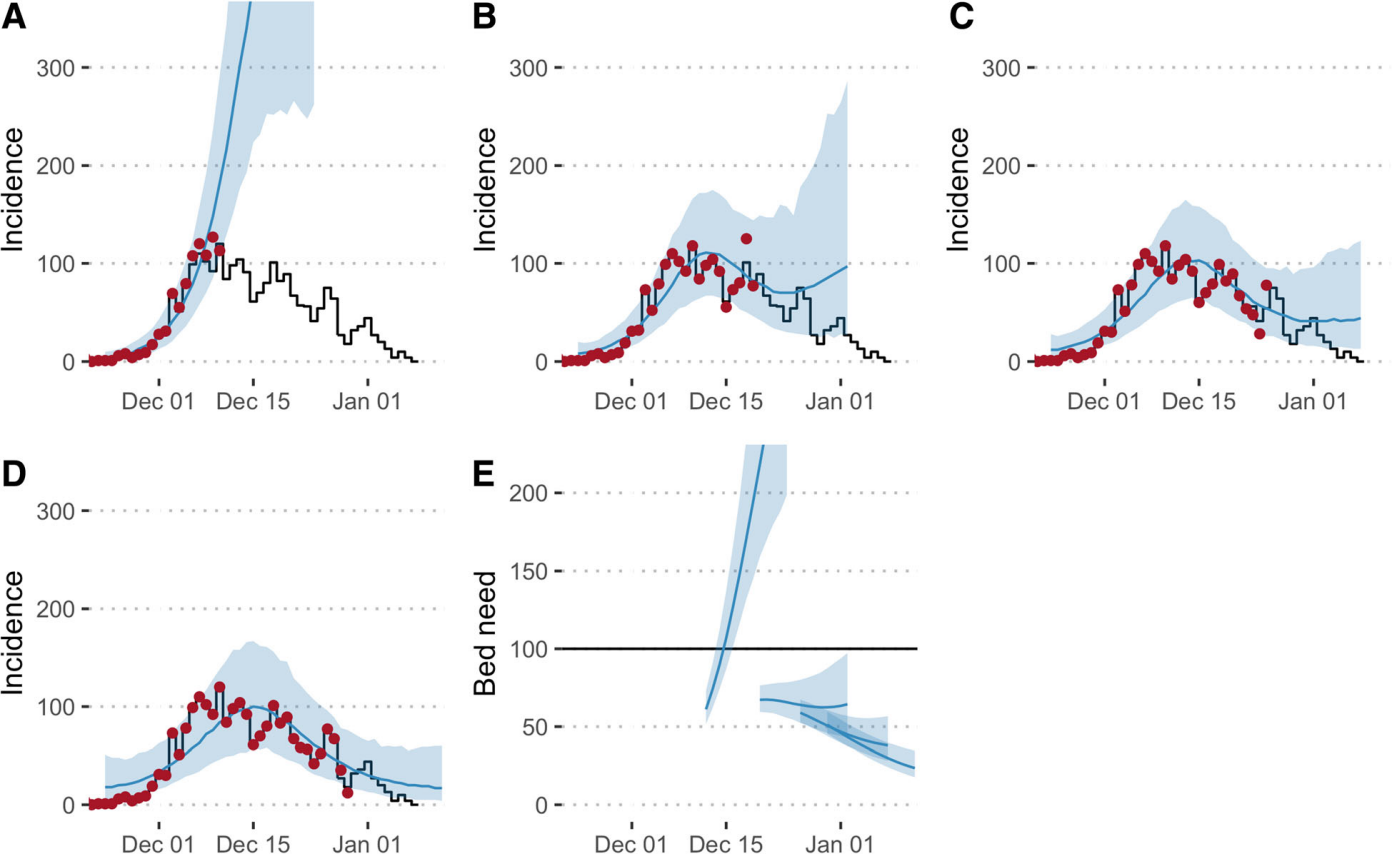
Total incidence over all age groups and locations (**a**–**d**) and bed need as forecasted by the model. Black lines show data as reported by 12 January 2018, red dots the adjusted incidence and blue lines and shaded areas the median and 2.5% and 97.5% percentiles according to 1000 model runs forecasting from 12 December (**a**), 20 December (**b**), 26 December (**c**) and 30 December (**d**). Forecasts of bed need issued on the same dates (**e**). The horizontal line shows the number of beds provided as of a decision taken on 14 December

**Fig. 4:**
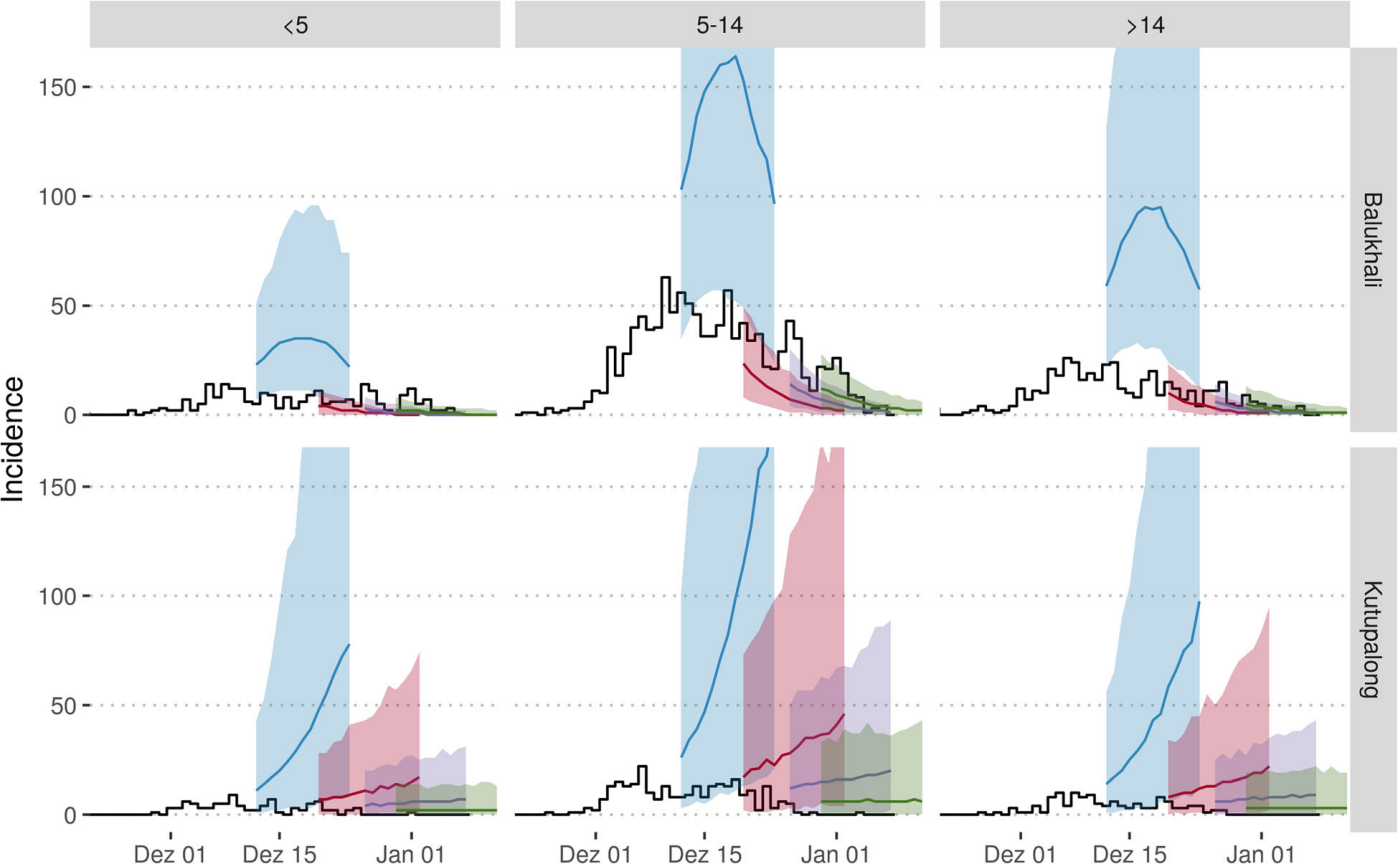
Incidence by location (rows) and age group (columns) as forecasted by the model. Black lines show data as reported by 12 January 2018, and coloured lines and shaded areas the median and 2.5% and 97.5% percentiles according to 1000 model runs forecasting from 12 December (blue), 20 December (red), 26 December (purple) and 30 December (green)

We re-simulated from the real-time model and retrospectively compared these outputs to the final recorded data (Fig. 3). At the first forecast timepoint, the model estimated that the epidemic would peak on 19 December in Balukhali with 303 (95% PPI 122–599) cases and would continue to grow beyond the 2-week forecast horizon in Kutupalong (Fig. 4). The required bed capacity at the end of the forecast horizon was estimated to be 316 (95% PPI 197–499). In reality, the peak of the epidemic (131 reported cases, 94 of which in Balukhali) had already occurred on 10 December, although it was not possible to conclude this from the data available in real time. On 19 December, a total of 54 cases were to be reported in Balukhali, lower than forecasted.

**Fig. 5:**
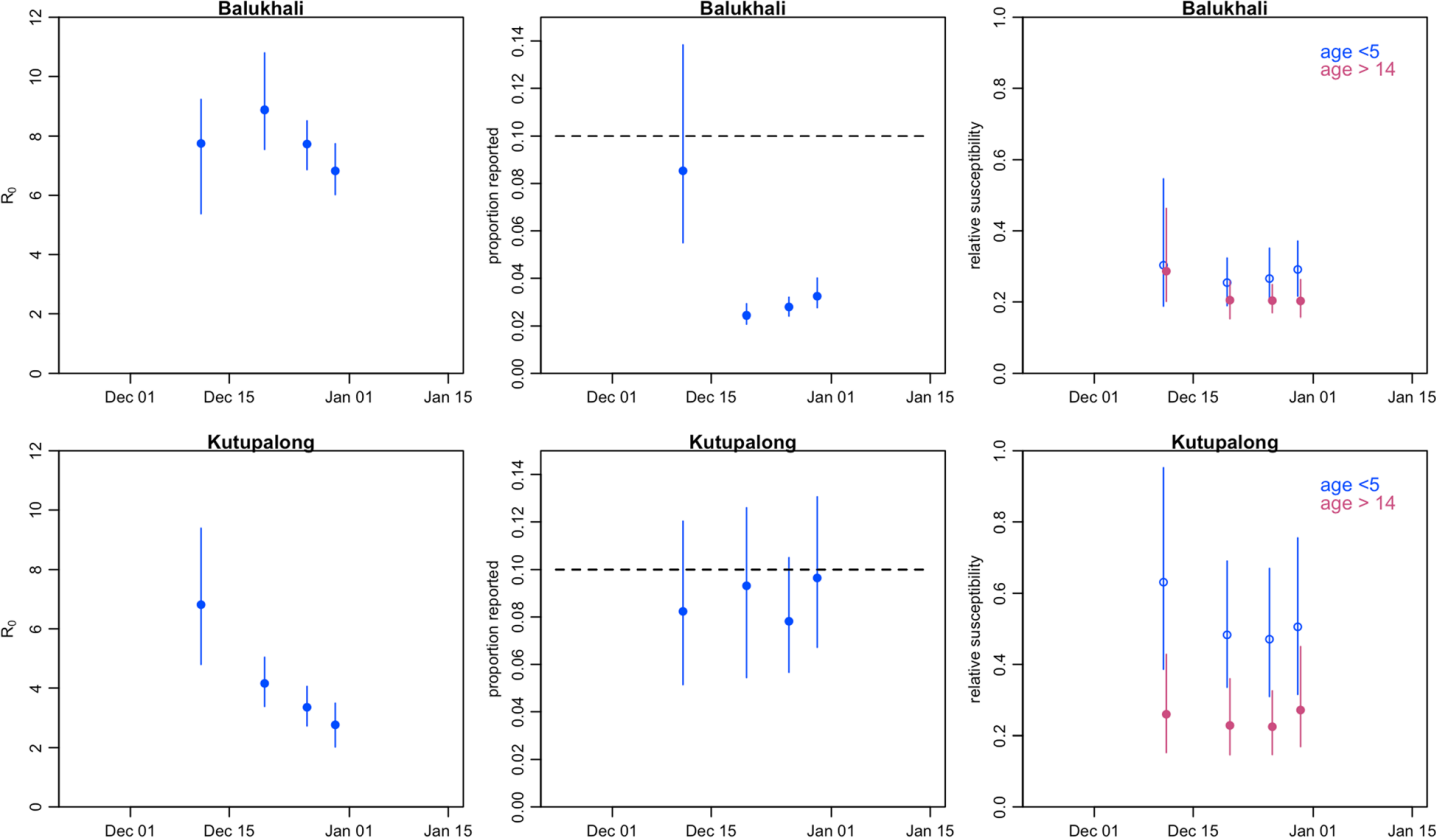
Posterior parameter values. Posterior ranges (vertical lines) and median values taken by model parameters for forecasts done on 12 December, 20 December, 26 December, 30 December and 8 January. The horizontal dashed lines show the mean value of the prior used for the proportion of reported. Uniform priors were used for other parameters

Forecasts became more accurate later during the epidemic. On 20 December, the model predicted a total of 912 cases (95% PPI 367–2183, this corresponds to 136 hospitalized patients under our assumptions), and on 26 December, a total of 270 cases (95% PPI 124–583, this corresponds to 41 hospitalized cases) up to the end of the year. In reality, 616 and 252 cases were actually reported during those respective periods. According to our model, *R*_0_ was equal to 7.8 (95% PPI 6.2–9.4) in Balukhali and 6.4 (95% PPI 4.4–8.9) in Kutupalong based on the initial forecast. Estimates later stabilized at lower values of 6.9 (95% PPI 6.1–7.7) and 2.8 (95% PPI 2.1–3.5) respectively on 26 December. The proportion of cases reported was estimated to be below the assumed prior median value of 10%, significantly so in Balukhali (Fig. 5). In both camps, susceptibility in the under 5 and over 15 age groups was estimated to be at least 50% lower than susceptibility in the 5–14 age group (Fig. 5).

In the model used in real time, we imposed an informative prior on the proportion of cases reported. To examine the sensitivity of our results to this assumption, we recalibrated our model with flat priors on reporting (Additional file 3: Figures S10–S16). Without the prior on reporting proportion, the initial forecast estimated that the epidemic had already peaked. However, this model fit was highly dependent on the apparent turnover suggested by the most recent fitted data point (Additional file 3: Figure S10). It also resulted in an underestimate of the later portion of the epidemic. Subsequent forecasts were better at capturing dynamics, but still tended to underestimate incidence. The model with a flat prior suggested that 3.0% (2.6–3.5%) of cases were reported in Balukhali and 0.35% (95% CrI 0.29–0.43%) in Kutupalong (Additional file 3: Figure S12).

### Operations and decision-making

The forecasts contributed to an evidence base that helped support operational aspects of the response, as well as advocacy for control measures. During December, staffing was increased in response to the outbreak. MSF employed a strategy of surge staffing for international staff and expedited recruitment of national staff doctors and nurses. On 17 December, a conservative decision to make a total of 100 hospital beds available was taken by MSF, using the high number of potential cases forecasted by the real-time modelling to guide the decision (with a view to monitor the modelling outputs over the coming weeks). It was also decided to categorize beds into two severity levels depending on clinical signs [22] and to treat mild cases in the community, which helped ensure that the available number of beds was never exceeded. Efforts to trace contacts of patients were intensified. Stocks of diphtheria antitoxin, which was in a global shortage due to other outbreaks in Yemen, Venezuela, Indonesia and Haiti at the time, antibiotics and other supplies were increased.

Initial advocacy for vaccination had centred on a broad age group. The modelling analysis highlighted that the under 5 and over 14 age groups were less susceptible relative to the 5–14 age group. As a result, the 5–14-year-old group contributed most to disease transmission, likely as a result of lack of vaccination in the displaced population before their arrival in Bangladesh. The discussions around forecasting also contributed to the advocacy to scale up outbreak response by other actors, such as the Global Outbreak Alert and Response Network, Samaritan’s Purse and the UK’s Emergency Medical Team, and helped lead to a closer collaboration between key partners such as MSF and WHO.

## Discussion

In this study, we have shown how transmission dynamic models and forecasting techniques provided insights into the epidemiological processes underlying the diphtheria outbreak in forcibly displaced Myanmar nationals living in camps and makeshift settlements in Cox’s Bazar district, Bangladesh. This enabled real-time analysis to estimate the course of the outbreak and corresponding resource needs.

Although our model captured the overall dynamic of the epidemic, there were several limitations to the modelling analysis. A number of key epidemiological parameters were unknown and had to be assumed from the literature or inferred from incidence data. In addition, the adjustment for reporting delays was initially biased upwards as delays between onset and case presentation shortened significantly during the early epidemic. These factors, combined with parameter uncertainty and a rapid increase in cases, led to the first forecast overestimating the number of future cases and made it difficult to capture the dynamics in Kutupalong.

Another limitation in the early stages of the analysis was that some key epidemiological unknowns could not be estimated individually from the data. During the initial exponential growth phase of an epidemic, it is generally not possible to estimate the marginal posteriors for all key unknown transmission and reporting parameters without imposing priors on at least some of them [23]. We therefore constrained prior susceptibility and the proportion of cases reported. Such assumptions, combined with remaining uncertainty about unknown parameters, can lead to substantial variability in forecast trajectories and potential bias in model outputs. In our real-time analysis, we fixed the proportion of cases susceptible and natural history parameters—equivalent for Dirac delta priors—and imposed a strong prior on the proportion of cases reported. In addition, our choice to impose an informative prior on the reporting rate was driven by the uncertainty surrounding the recent incidence data points in real time. Owing to the initially very long reporting delays, we were aware that the newest raw data could give a false impression of a declining epidemic (Fig. 2), creating the risk of substantially underestimating epidemic magnitude in our forecasts.

To retrospectively assess the sensitivity of our results to the prior assumptions, we recalibrated our model with flat priors on reporting, leading to lower estimates of the reporting rate (Additional file 3: Figure S12). The posterior estimates from the model used in real time reflected this lower reporting rate by 26 December for Balukhali but remained close to the informative prior for Kutupalong (Fig. 5). Although in retrospect the forecasts using flat priors capture the outbreak dynamics well and anticipate the epidemic peak, it would have been difficult to confidently conclude the epidemic had peaked in real time, given that such a conclusion would be heavily reliant on very recent data points, which were known to be less reliable (e.g. Fig. 3b). With more time available, it would have been possible to explore the implications of our prior assumptions by running multiple models with different priors for different scenarios in real time, comparing results and accompanying uncertainty. Note, however, that it would still have been possible to conclude which model performs best only a posteriori because of the abovementioned uncertainty regarding the long reporting delays and the data about the further course of the outbreak not yet being available.

Our model did not capture time variations in key parameters such as the reproduction number (i.e. due to interventions, such as contact tracing and active case finding or the WHO-lead vaccination campaign initiated on 12 December 2017) or the reporting rate (e.g. due to changes in health-seeking behaviour induced by health promotion activities and circulating information about the outbreak itself). The introduction of vaccination is, however, unlikely to have had an impact within the time frame analysed here given the delay to protection, incubation period following infection and the delay in reporting following onset.

Further, we assumed a fixed population size. In reality, there can be a substantial influx of people into camps during outbreaks, as well as movement within and between camps; understanding how such movements might affect outbreak dynamics in general would be worth investigating in future studies.

In addition to the limitations mentioned above, the deterministic model we used attributed any uncertainty to the fitted parameters and the reporting process, rather than stochasticity in transmission. This study focuses on reporting the performance of our model used in real time. With the benefits of hindsight, we could nevertheless have considered a number of adaptations to our model. A stochastic model could have been used to include a more accurate representation of uncertainty, in addition to capturing unexplained time variations in parameters and transmission rate and thus allowing for turnover due to other factors than depletion of susceptibles [13, 24, 25]. A better representation of the reporting process, for instance explicitly taking account of under-reporting of mild cases seeking primary care with one of the numerous health facilities run by various organizations [26] or correcting for a potential spatial bias in reporting [27], could have been beneficial to decrease uncertainties related to the case to infection ratio. In addition, the correction for the reporting delay could have been explicitly included within the model in order to better capture the associated uncertainty. A more complex model, however, would have been more time consuming to set up and calibrate and would still have been reliant on imperfect data, responsible for large parts of the remaining uncertainty about the epidemiological processes and the model output. More generally, questions of which modelling approaches work best in which outbreak situations and for which diseases should be addressed between outbreaks, as part of routine research.

Our estimated values for the basic reproduction number *R*_0_ were in agreement with values from the literature [7, 28] and other estimates for the same epidemic [29], although our assumed generation times were lower and estimates of the reporting rate were higher compared to an analysis of the early diphtheria outbreak dynamics by Matsuyama et al. [29], who did not stratify by age or camp. Whereas we assumed that the susceptibility was greatest in the age group of 5–14 years, the proportion of cases in the age group of 15 years and above was higher before the epidemic peak than after. This may indicate either that adults made a substantial contribution to transmission during the epidemic growth phase [30] or that relative age-specific reporting changed during the course of the outbreak.

Construction of mechanistic epidemic models makes it possible to formalize assumptions about the epidemiological processes underlying an outbreak, incorporating expert knowledge and context-specific analysis of the local situation. When working in real time, the main challenge lies in quickly consolidating all necessary information—in an often complex and variable emergency situation—to be able to make appropriate assumptions in a model. In our case, a better understanding of epidemiological processes, disease characteristics, case reporting and prior vaccination status would have allowed for more accurate assumptions and potentially more accurate forecasts. Despite regular discussions between LSHTM and MSF during December, and as a result of the slow start of the epidemic, the first forecasts were only delivered a month after the first case was reported.

Our experiences of real-time modelling and analysis during this outbreak highlighted the importance of effective ongoing communication with field staff. Besides enabling access to real-time data (including incidence, demography and geography), staff can also provide additional context and information such as the general epidemiological situation, likely vaccination status of the population, nature and severity of symptoms, health-seeking behaviour and access to health care, sanitary situation and population movements. To maximize the future benefit of real-time modelling, it would be advantageous to build strong, long-term collaborations between organizations providing outbreak responses and epidemiological modellers [31]. Such collaborations should focus on establishing well-defined processes (i.e. analysis pipelines) on how to collect, treat and share relevant data and other information from the field with modellers, ideally embedding an experienced modeller or data manager in the outbreak response team and enabling model results and model-based recommendations to be fed back to field staff and decision-makers, whose input can in turn inform subsequent analysis.

## Conclusions

Although modelling is only one component of the evidence base for decision-making in outbreak situations, we have shown that suitable analysis and forecasting techniques can be used to gain insights into an ongoing outbreak.

In the context of the diphtheria outbreak in Bangladesh, real-time modelling made it possible to feedback key information about the potential scale of the epidemic, likely resource needs and underlying mechanisms of transmission to decision-makers at a time when this information was largely unknown. By 20 December, our model was able to generate reliable forecasts with a lead time of 2 weeks.

We propose that such analysis can be further developed in the future through strengthening collaborations and setting up bi-directional data and information flow pipelines linking modellers with decision-makers and field staff, so that real-time modelling can rapidly and routinely contribute to outbreak response.

## Additional files

Additional file 1: Population data. (CSV 82 bytes)

Additional file 2: Incidence data - The unadjusted incidence data extracted from line lists and used for this analysis. (CSV 642 kb)

Additional file 3: **Figure S1.** Early case onset reports. **Figure S2.** Posterior parameter values 12th December 2018. **Figure S3.** Posterior parameter values 20th December 2018. **Figure S4.** Posterior parameter values 26th December 2018. **Figure S5.** Posterior parameter values 30th December 2018. **Figure S6.** MCMC chains for 12th December 2018. **Figure S7.** MCMC chains for 20th December 2018. **Figure S8.** MCMC chains for 26th December 2018. **Figure S9.** MCMC chains for 30th December 2018. **Figure S10.** Forecasts assuming flat prior on reporting. **Figure S11.** Incidence forecasts by age and location assuming flat prior on reporting. **Figure S12.** Posterior parameter values assuming flat prior on reporting. **Figure S13.** MCMC chains for 12th December 2018 assuming flat prior on reporting. **Figure S14.** MCMC chains for 20th December 2018 assuming flat prior on reporting. **Figure S15.** MCMC chains for 26th December 2018 assuming flat prior on reporting. **Figure S16.** MCMC chains for 30th December 2018 assuming flat prior on reporting. (PDF 3540 kb)

## Abbreviations

DTC: Diphtheria Treatment Centre
LSHTM: London School of Hygiene and Tropical Medicine
MCMC: Markov Chain Monte Carlo
MSF: Médecins sans Frontières
WHO: World Health Organization
