# Supplementary material for: Real-time analysis of the diphtheria outbreak in forcibly displaced Myanmar nationals in Bangladesh
Source: BMC Med. 2019 Mar 12;17:58. doi: 10.1186/s12916-019-1288-7 (PMC6413455; doi:10.1186/s12916-019-1288-7)
Supplement: Supplementary file 3 — Figure S1. Early case onset reports. Figure S2. Posterior parameter values 12th December 2018. Figure S3. Posterior parameter values 20th December 2018. Figure S4. Posterior parameter values 26th December 2018. Figure S5. Posterior parameter values 30th December 2018. Figure S6. MCMC chains for 12th December 2018. Figure S7. MCMC chains for 20th December 2018. Figure S8. MCMC chains for 26th December 2018. Figure S9. MCMC chains for 30th December 2018. Figure S10. Forecasts assuming flat prior on reporting. Figure S11. Incidence forecasts by age and location assuming flat prior on reporting. Figure S12. Posterior parameter values assuming flat prior on reporting. Figure S13. MCMC chains for 12th December 2018 assuming flat prior on reporting. Figure S14. MCMC chains for 20th December 2018 assuming flat prior on reporting. Figure S15. MCMC chains for 26th December 2018 assuming flat prior on reporting. Figure S16. MCMC chains for 30th December 2018 assuming flat prior on reporting. (PDF 3540 kb) [file 12916_2019_1288_MOESM3_ESM.pdf]

Supplementary Figures

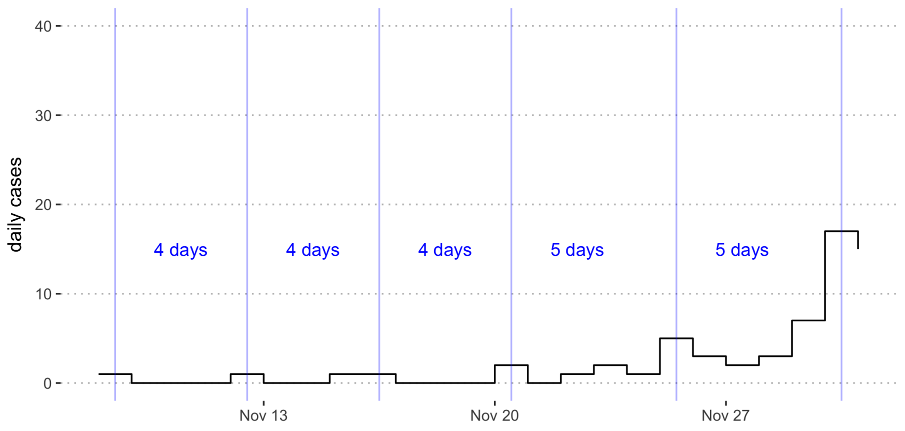

Figure S1: Early case onset reports. Peaks in cases typically occurred 4–5 days apart, potentially reflecting generations of infection. Data shown from line list dated 6th December 2017.

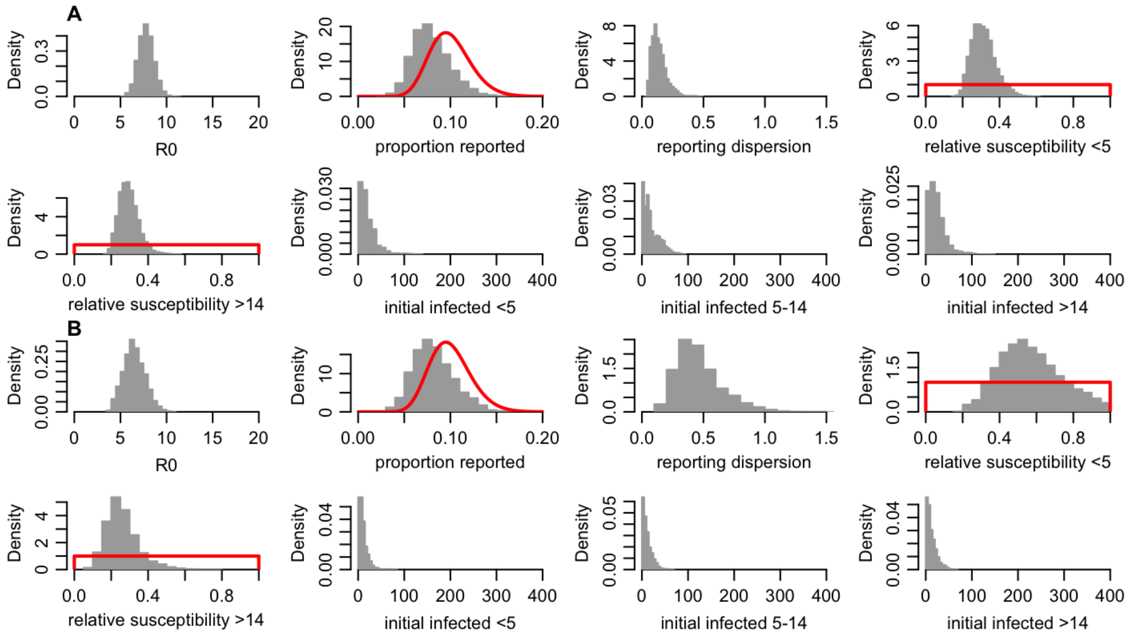

Figure S2: Posterior parameter values from model used to produce forecasts on 12th December 2018. (A) Balukhali, (B) Kutupalong. Red lines show prior distributions.

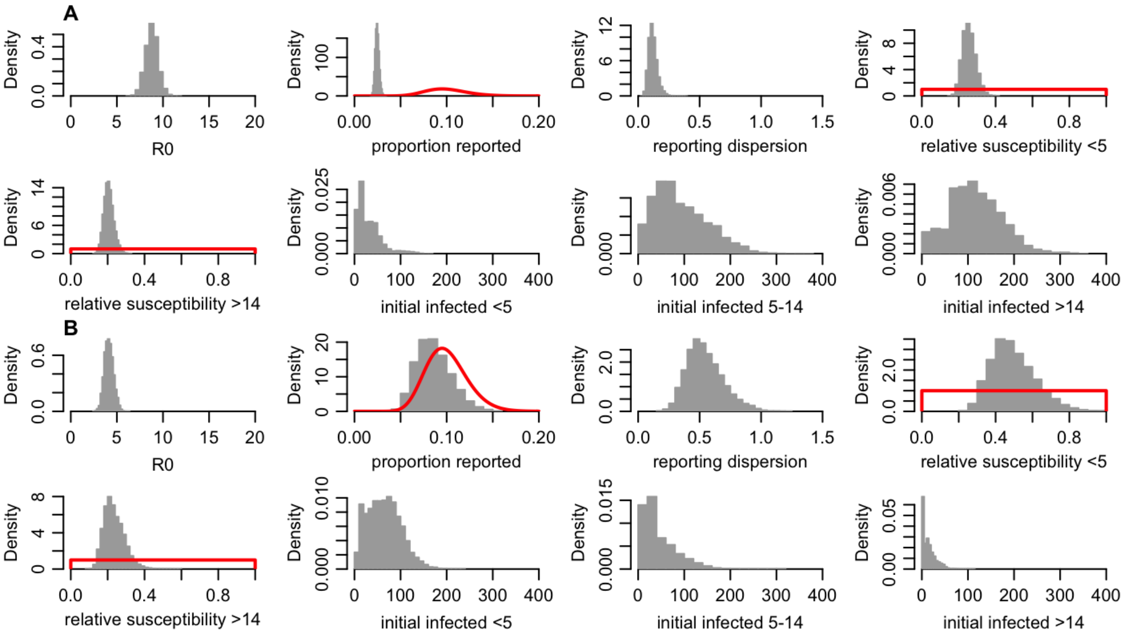

Figure S3: Posterior parameter values from model used to produce forecasts on 20th December 2018. (A) Balukhali, (B) Kutupalong. Red lines show prior distributions.

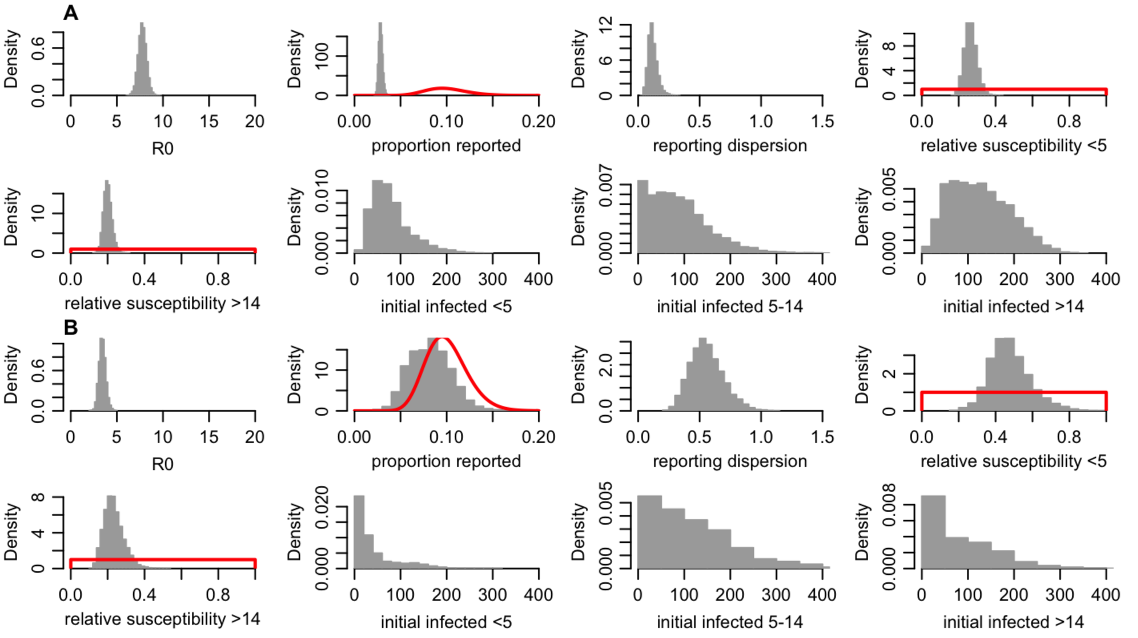

Figure S4: Posterior parameter values from model used to produce forecasts on 26th December 2018. (A) Balukhali, (B) Kutupalong. Red lines show prior distributions.

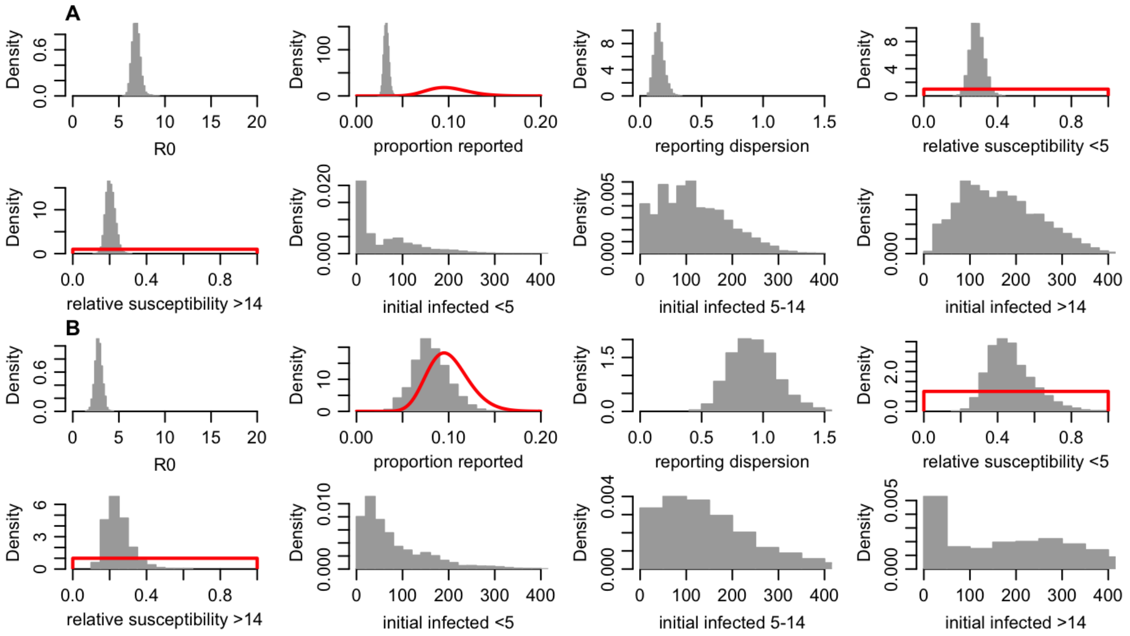

Figure S5: Posterior parameter values from model used to produce forecasts on 30th December 2018. (A) Balukhali, (B) Kutupalong. Red lines show prior distributions.

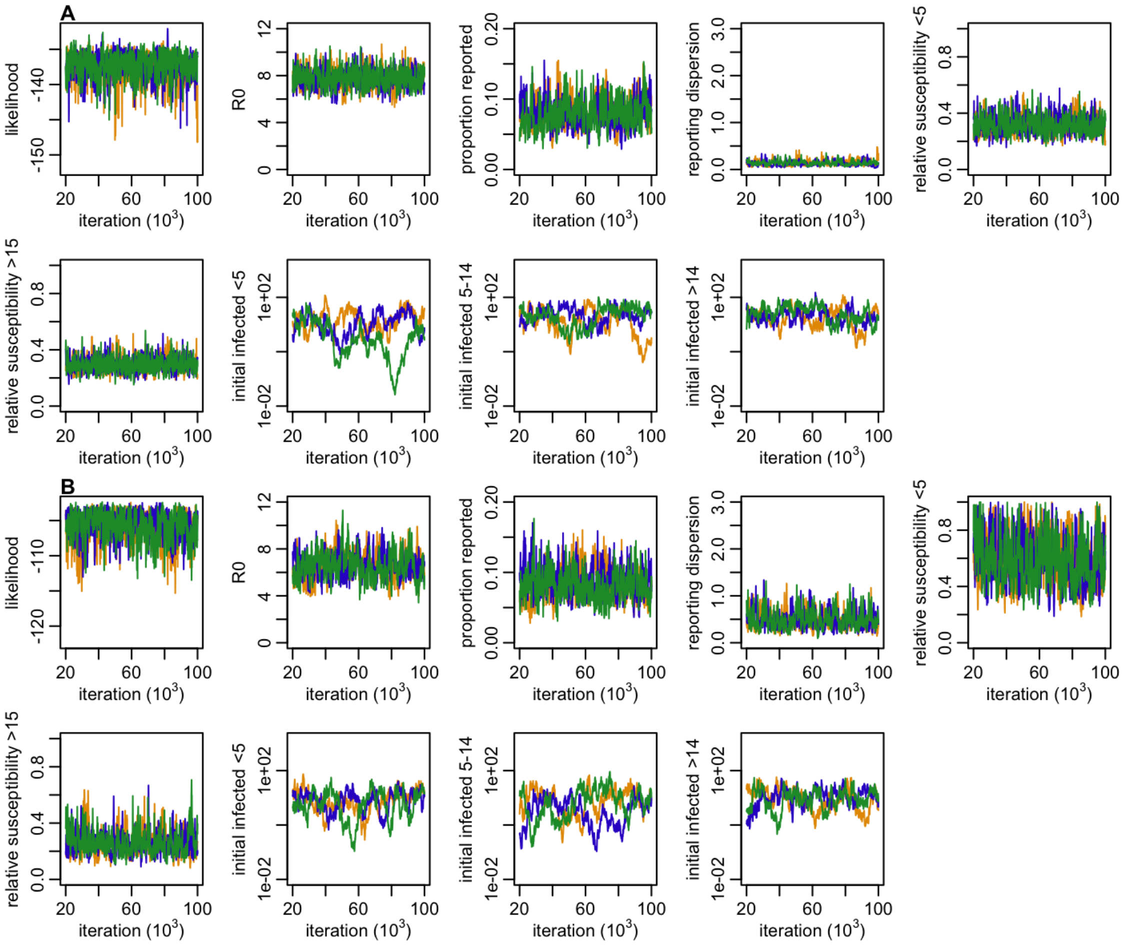

Figure S6: MCMC chains from model used to produce forecasts on 12th December 2018. (A) Balukhali, (B) Kutupalong. Each line shows a separate MCMC chain, within the initial burn-in period omitted.

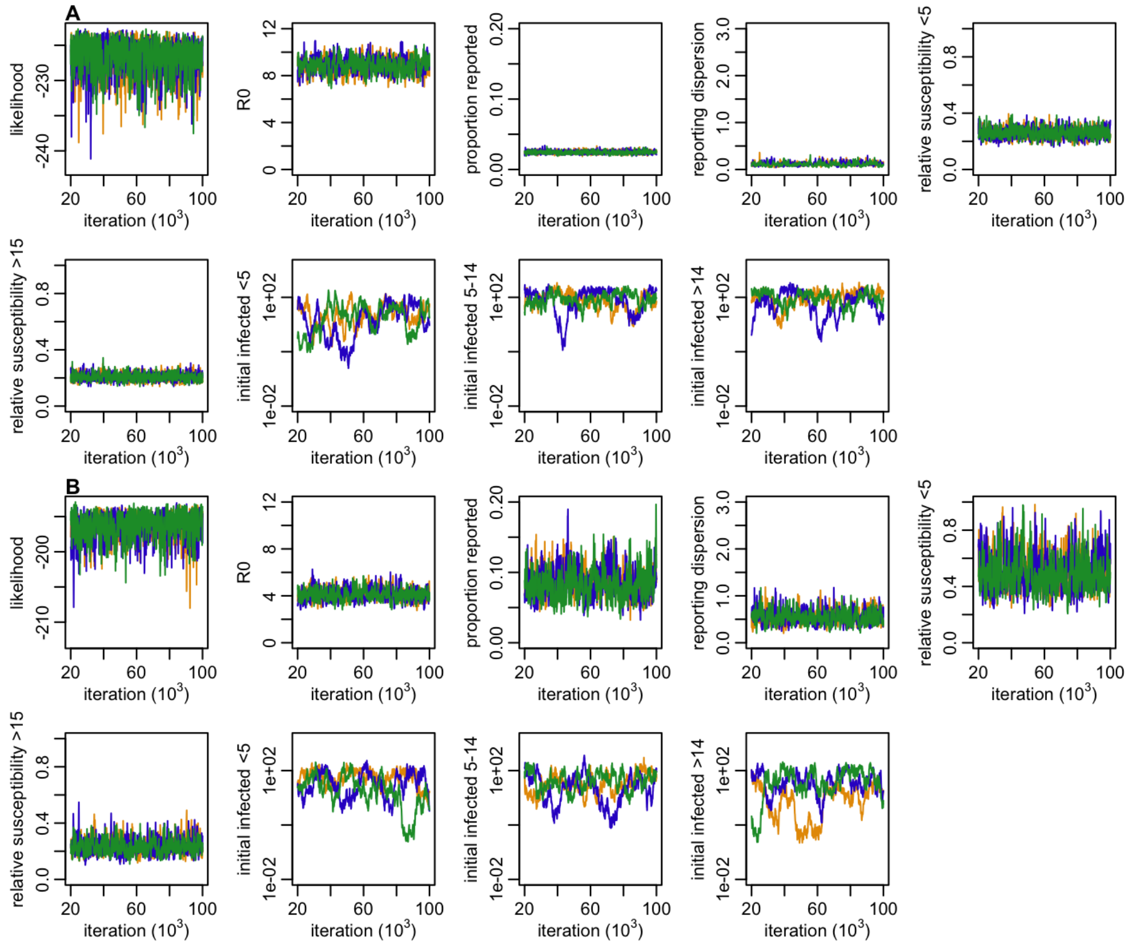

Figure S7: MCMC chains from model used to produce forecasts on 20th December 2018. (A) Balukhali, (B) Kutupalong. Each line shows a separate MCMC chain, within the initial burn-in period omitted.

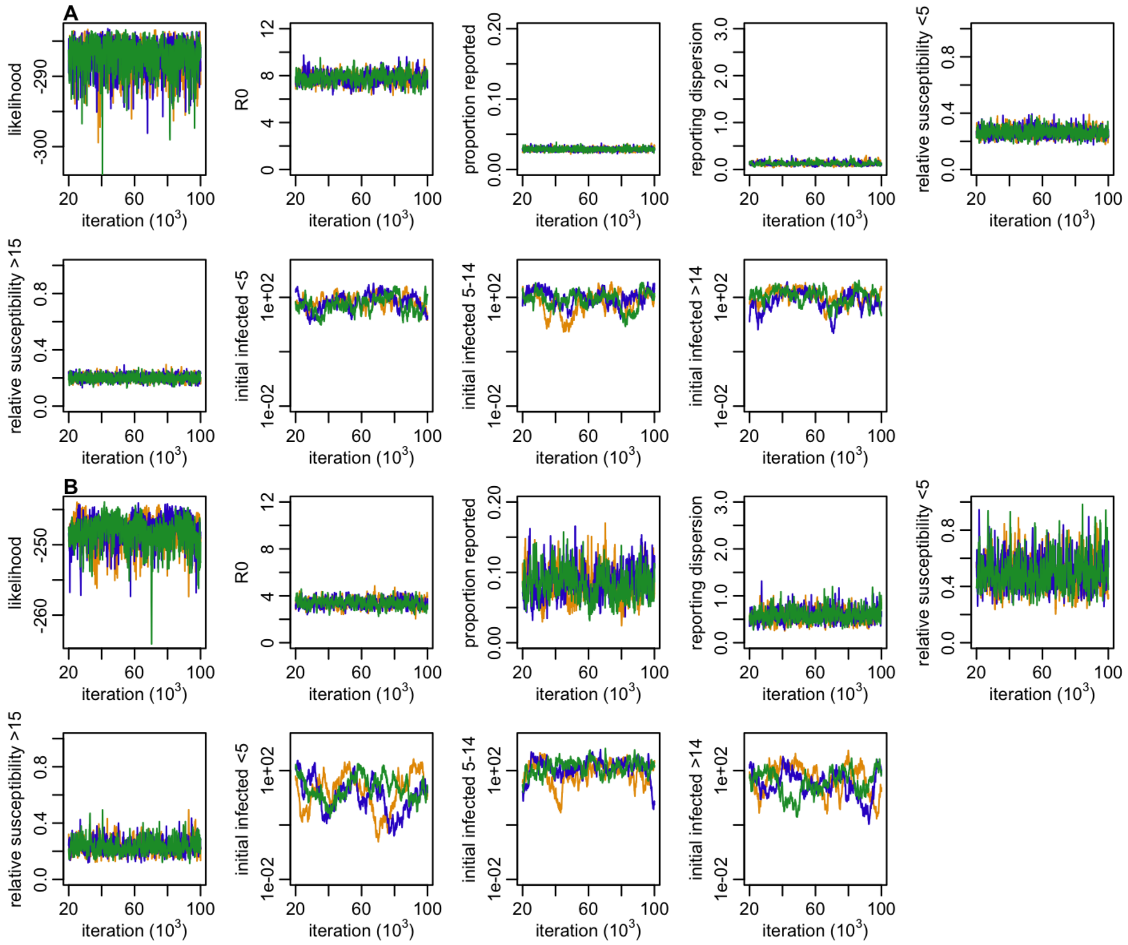

Figure S8: MCMC chains from model used to produce forecasts on 26th December 2018. (A) Balukhali, (B) Kutupalong. Each line shows a separate MCMC chain, within the initial burn-in period omitted.

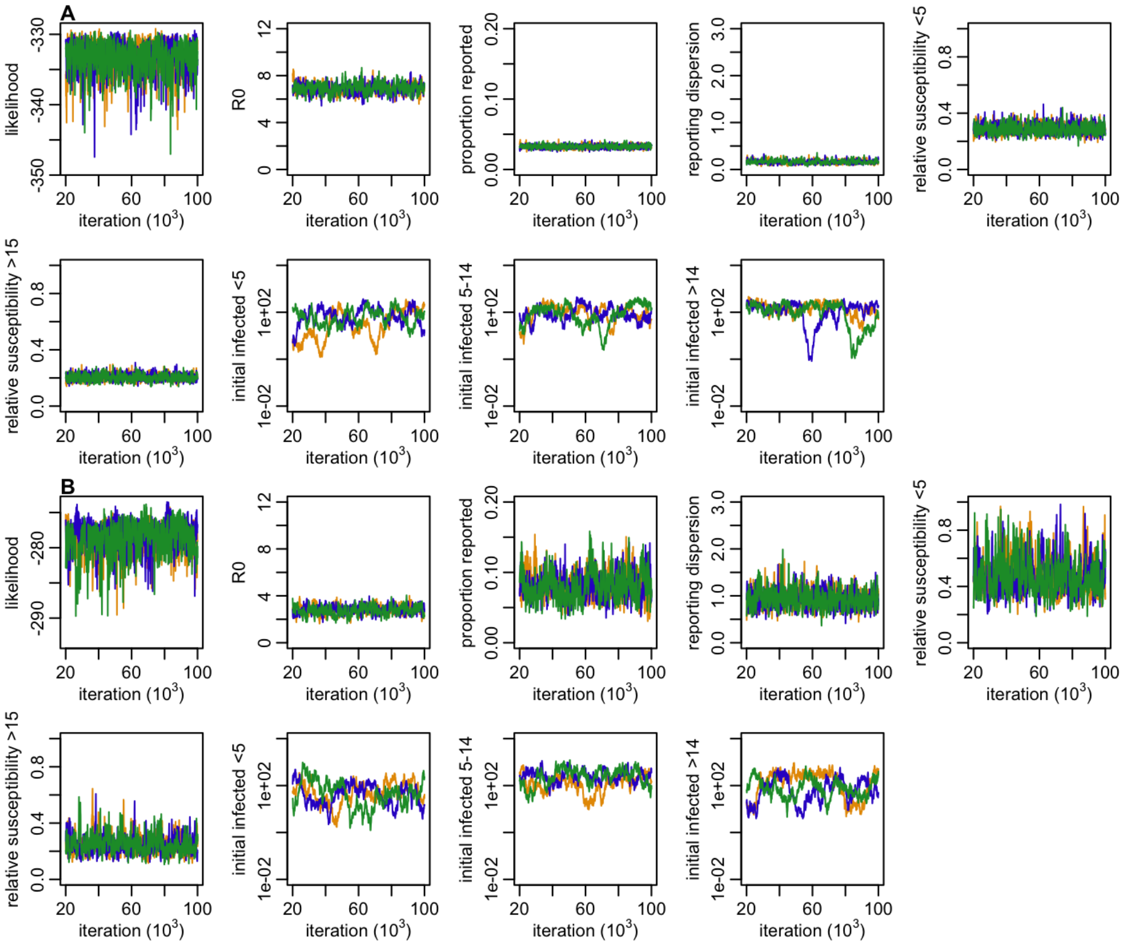

Figure S9: MCMC chains from model used to produce forecasts on 30th December 2018. (A) Balukhali, (B) Kutupalong. Each line shows a separate MCMC chain, within the initial burn-in period omitted.

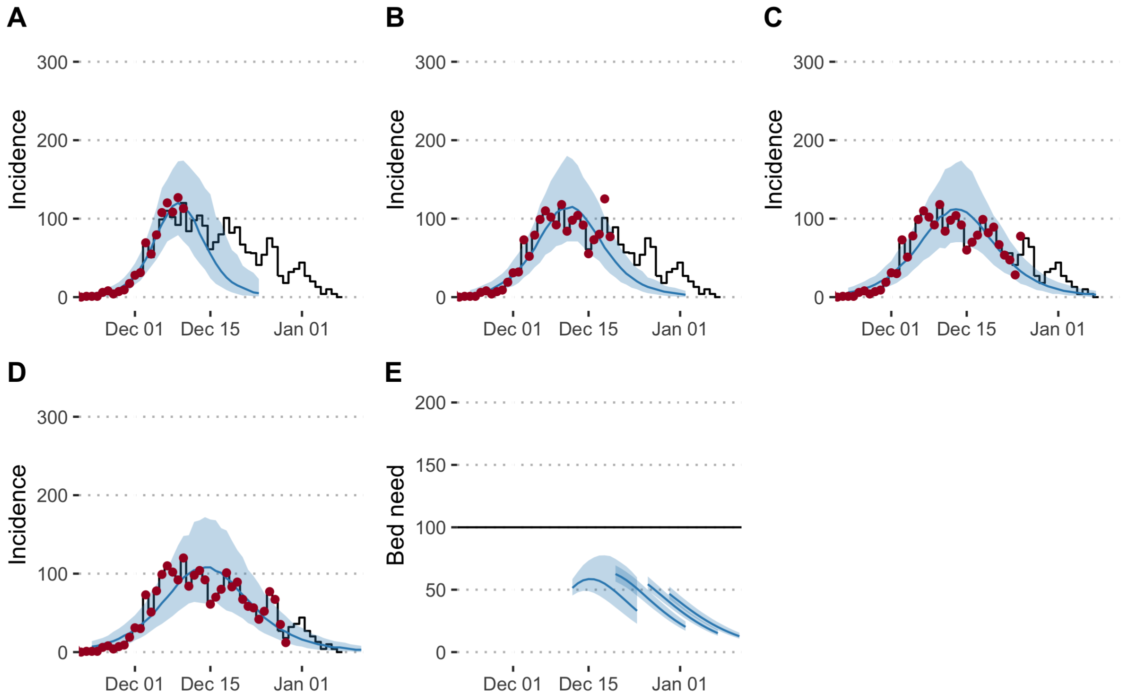

Figure S10: Total incidence over all age groups and locations (A-D) and bed need as forecasted by the model, assuming a flat prior on reporting proportion. Black lines show data as reported by 12 January 2018, red dots the adjusted incidence and blue lines and shaded areas the median and 2.5% and 97.5% percentiles according to 1000 model runs forecasting from 12th December (A), 20th December (B), 26th December (C) and 30th December (D). Forecasts of bed need issued on the same dates (E). The horizontal line shows the number of beds provided as of a decision taken on 14th December.

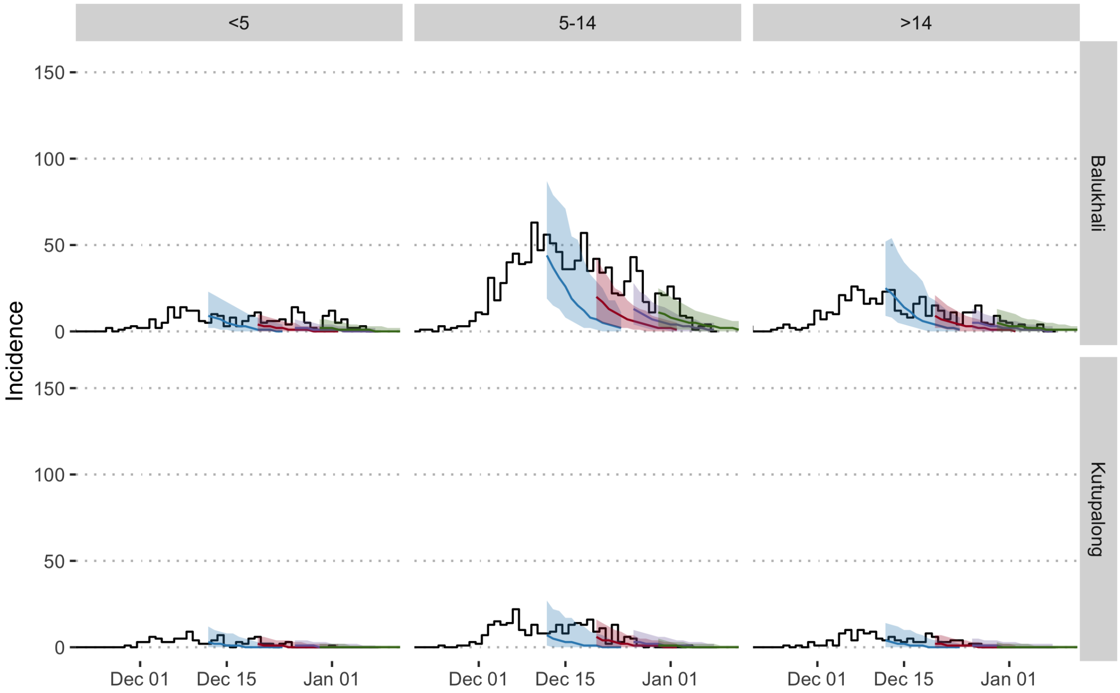

Figure S11: Incidence by location (rows) and age group (columns) as forecasted by the model, assuming a flat prior on reporting proportion. Black lines show data as reported by 12 January 2018 and coloured lines and shaded areas the median and 2.5% and 97.5% percentiles according to 1000 model runs forecasting from 12th December (blue), 20th December (red), 26th December (purple) and 30th December (green).

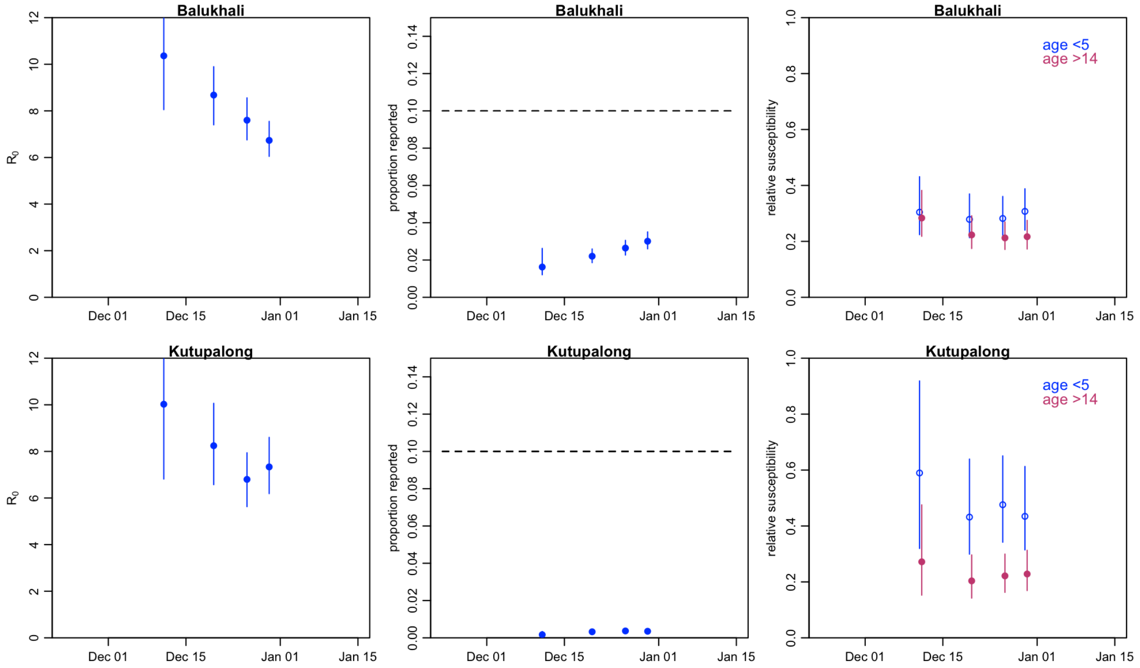

Figure S12: Posterior parameter values, assuming a flat prior on reporting proportion. Posterior ranges (vertical lines) and median values taken by model parameters for forecasts done on 12th December, 20th December, 26th December, 30th December and 8th January. The horizontal dashed lines show the mean value of the prior used for the proportion of reported. Uniform priors were used for other parameters.

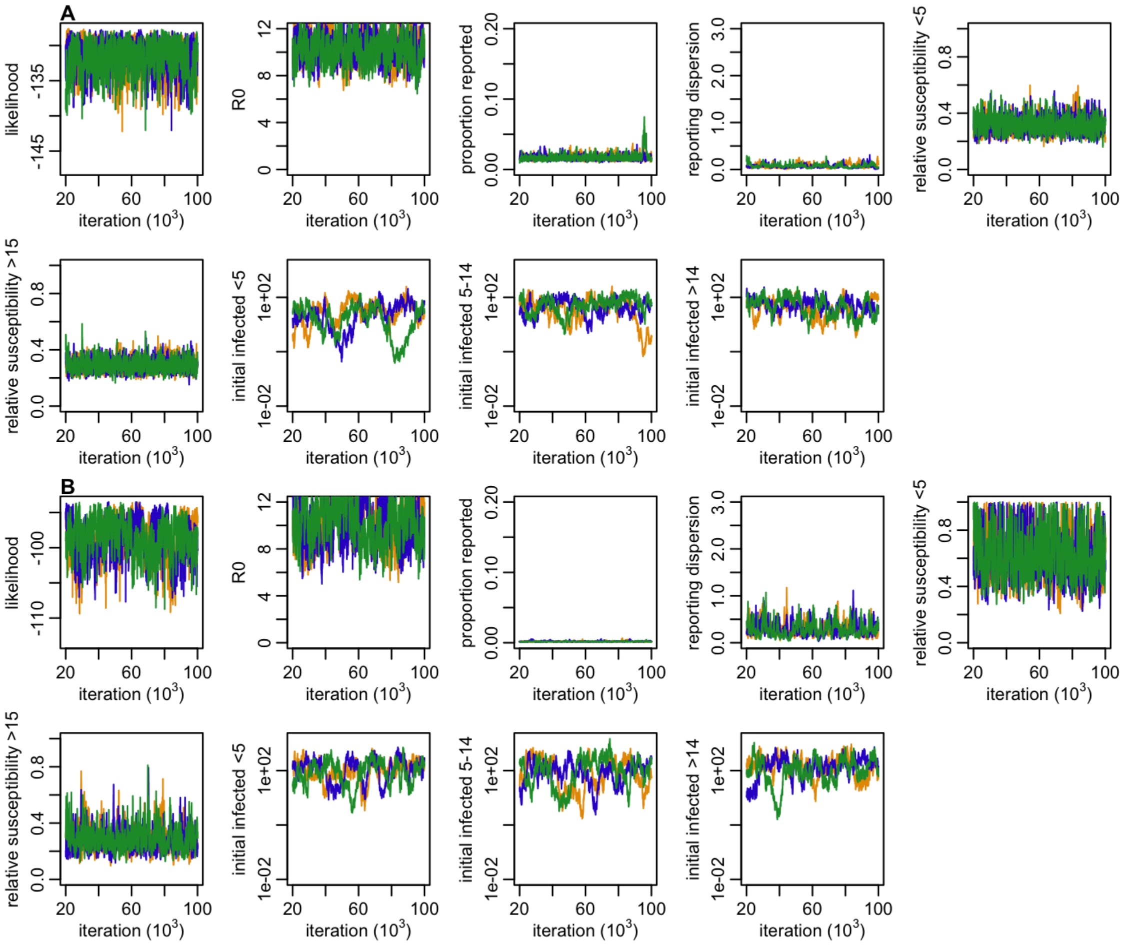

Figure S13: MCMC chains from model used to produce forecasts on 12th December 2018, assuming a flat prior on reporting proportion. (A) Balukhali, (B) Kutupalong. Each line shows a separate MCMC chain, within the initial burn-in period omitted.

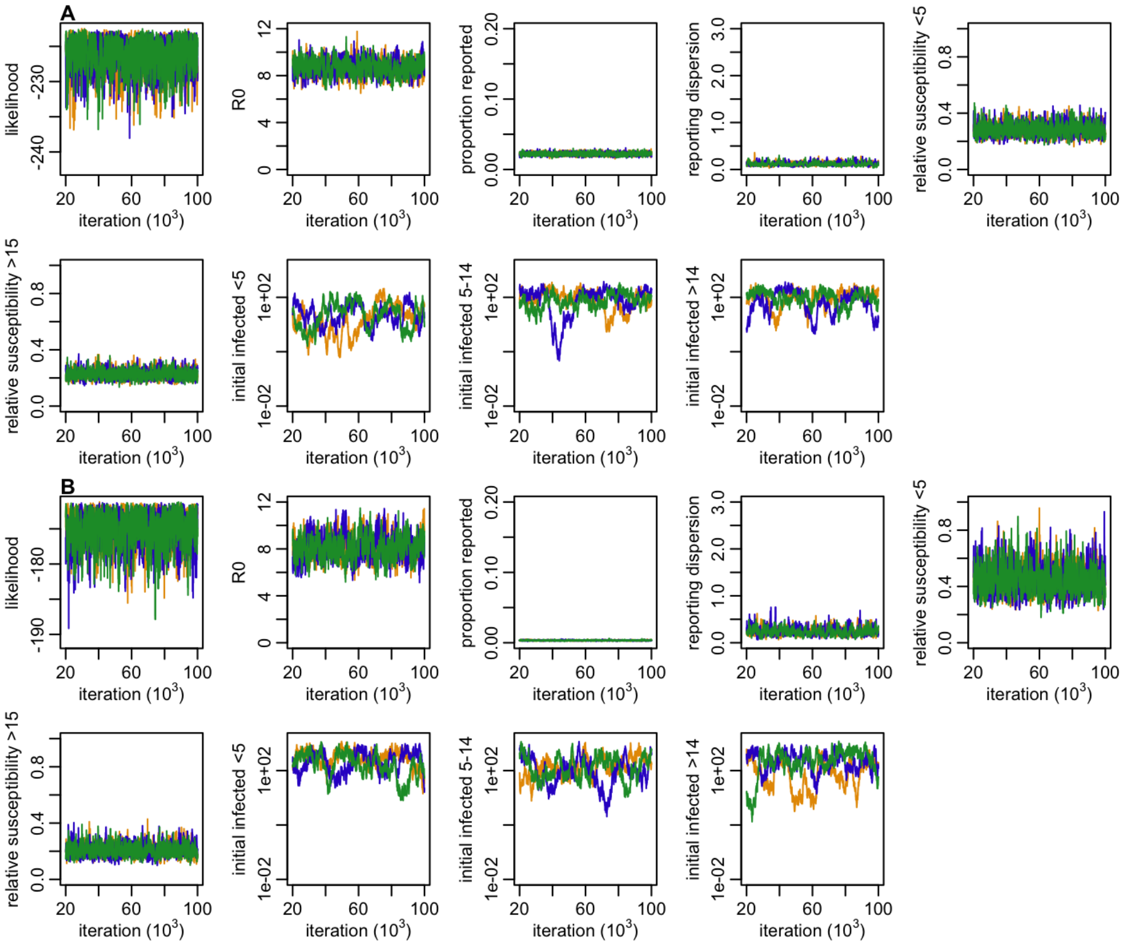

Figure S14: MCMC chains from model used to produce forecasts on 20th December 2018, assuming a flat prior on reporting proportion. (A) Balukhali, (B) Kutupalong. Each line shows a separate MCMC chain, within the initial burn-in period omitted.

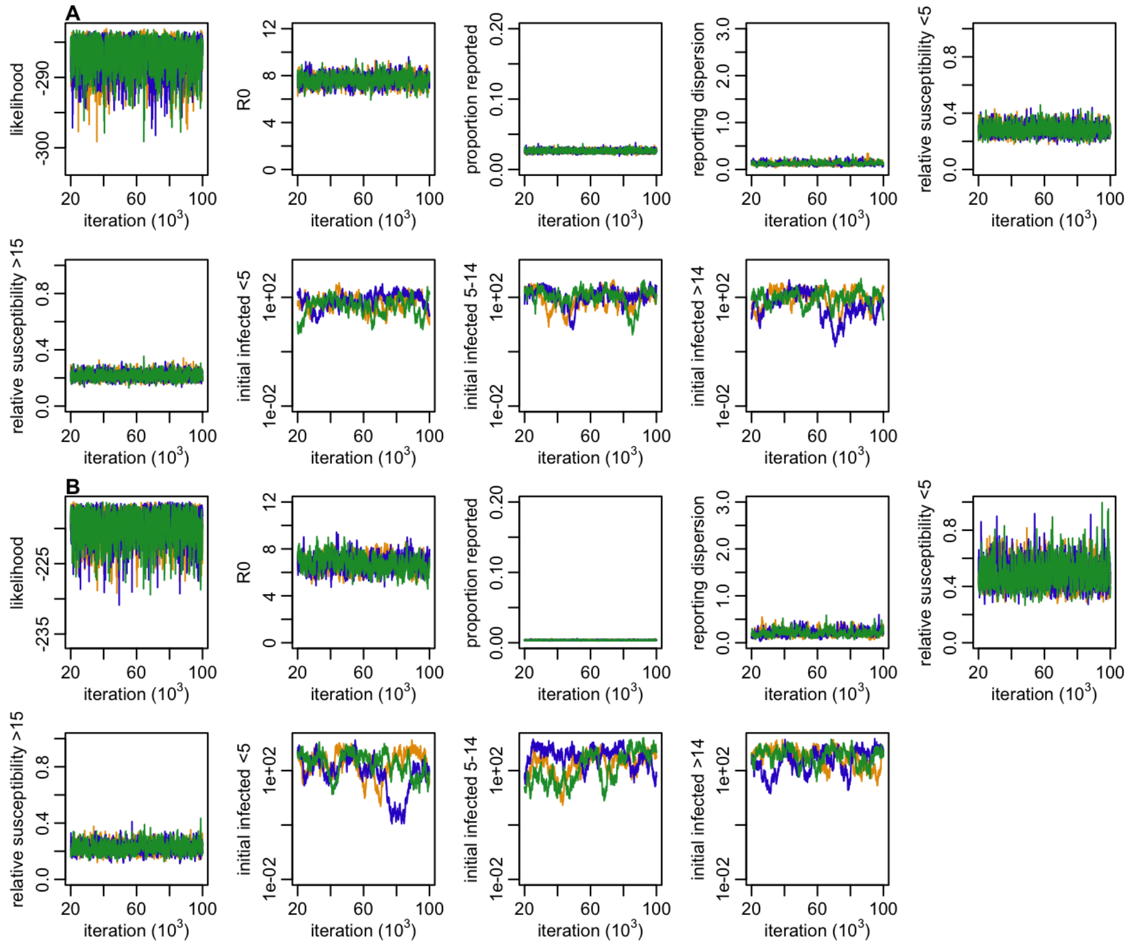

Figure S15: MCMC chains from model used to produce forecasts on 26th December 2018, assuming a flat prior on reporting proportion. (A) Balukhali, (B) Kutupalong. Each line shows a separate MCMC chain, within the initial burn-in period omitted.

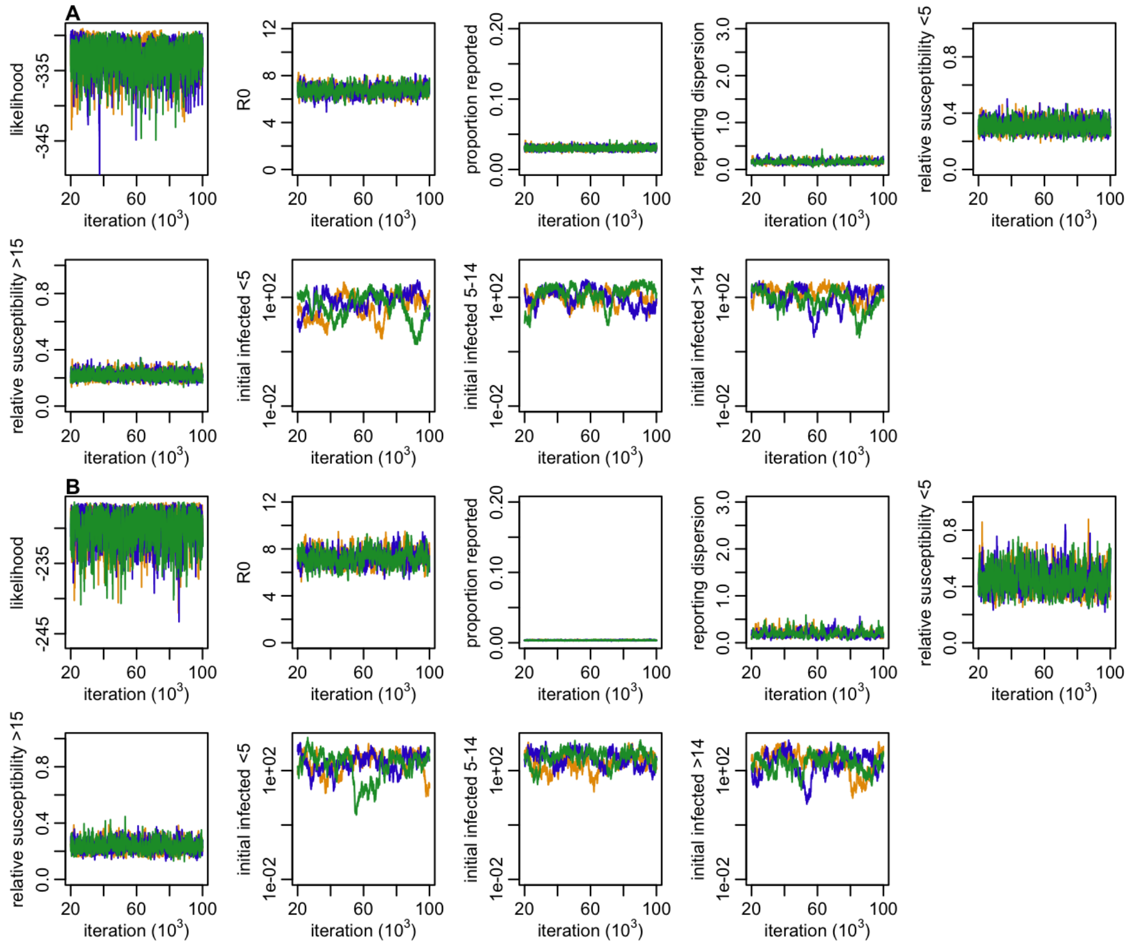

Figure S16: MCMC chains from model used to produce forecasts on 30th December 2018, assuming a flat prior on reporting proportion. (A) Balukhali, (B) Kutupalong. Each line shows a separate MCMC chain, within the initial burn-in period omitted.
